# Differential Evaluating Effect on Exercise Capacity of Cardiopulmonary Exercise Testing and Treadmill Exercise Testing in Post-percutaneous Coronary Intervention Patients

**DOI:** 10.3389/fcvm.2021.682253

**Published:** 2021-07-28

**Authors:** Yifan Gao, Bin Feng, Rong Hu, YingYue Zhang, Yajun Shi, Yong Xu, Jing Ma

**Affiliations:** ^1^Medical School of Chinese PLA, Beijing, China; ^2^Department of Cardiology, Chinese PLA General Hospital, Beijing, China

**Keywords:** cardiac rehabilitation, exercise test, coronary artery, exercise prescription, aerobic exercise

## Abstract

**Background:** Treadmill exercise testing (TET) is commonly used to measure exercise capacity. Studies have shown that cardiopulmonary exercise testing (CPET) is more accurate than TET and is, therefore, regarded as the “gold standard” for testing maximum exercise capacity and prescribing exercise plans. To date, no studies have reported the differences in exercise capacity after percutaneous coronary intervention (PCI) using the two methods or how to more accurately measure exercise capacity based on the results of TET.

**Aims:** This study aims to measure maximum exercise capacity in post-PCI patients and to recommend exercise intensities that ensure safe levels of exercise.

**Methods:** We enrolled 41 post-PCI patients who were admitted to the Cardiac Rehabilitation Clinic at the First Medical Center, the Chinese PLA General Hospital, from July 2015 to June 2016. They completed CPET and TET. The paired sample *t*-test was used to compare differences in measured exercise capacity, and multiple linear regression was applied to analyze the factors that affected the difference.

**Results:** The mean maximum exercise capacity measured by TET was 8.89 ± 1.53 metabolic equivalents (METs), and that measured by CPET was 5.19 ± 1.23 METs. The difference between them was statistically significant (*p* = 0.000) according to the paired sample *t*-test. The difference averaged 40.15% ± 2.61% of the exercise capacity measured by TET multiple linear regression analysis showed that the difference negatively correlated with waist-hip ratio (WHR).

**Conclusion:** For the purpose of formulating more accurate exercise prescription, the results of TET should be appropriately adjusted when applied to exercise capacity assessment.

**Clinical Trial Registration:**http://www.chictr.org.cn/ number, ChiCTR2000031543.

## Introduction

Reasonable cardiac rehabilitation (CR), especially exercise training can effectively reduce heart-related symptoms in post-percutaneous coronary intervention (post-PCI) patients, improve long-term prognosis, and reduce the probability of recurrent myocardial infarction or unscheduled revascularization (1–3). The most important aspect of exercise training is identification of appropriate exercise intensity depending a lot on maximum exercise capacity, so as to accurately instruct patients with cardiovascular disease how to exercise safely. There are many ways to measure exercise capacity, including the 6-min walk test and treadmill exercise testing (TET). When patients are instructed to return to daily life, levels of exercise capacity correspond to various daily activities (4–6). It has been revealed that cardiopulmonary exercise testing (CPET), a method that accurately measures oxygen and carbon dioxide metabolism, measures true oxygen uptake and estimates maximum exercise capacity more accurately. Therefore, it is considered the “gold standard” for testing maximum exercise capacity and prescribing exercise plans (7, 8).

However, in most developing countries, CPET is not common, and doctors had to use the results of ordinary TET to estimate the maximum exercise capacity, so as to formulate exercise prescriptions for post-PCI patients (9). In developed countries, despite the decline in the use of TET for clinical purposes in recent years, there are still examples of exercise capacity estimation through it (10). Actually, ordinary TET lacks synchronous gas metabolism monitoring and can only estimate the exercise capacity at various stages. Moreover, the results estimated by different TET protocols were inconsistent (11). It has been pointed out that exercise capacity estimated by TET is greater than that measured by CPET (12–14). Consequently, using TET results to formulate exercise prescription may pose a risk of recommending exercise intensity that exceeds the patient's actual capacity and increase the risk of cardiovascular events.

Nevertheless, the similarities and differences between CPET and TET in evaluating the maximum exercise capacity in the post-PCI patients remain largely unknown. An in-depth study on the conversion relationship between TET and CPET would provide accurate, objective values for medical institutions without CPET, thereby improving the safety of post-PCI patients during exercise training. Therefore, the present study employed CPET and TET to measure the maximum exercise capacity of post-PCI patients and to examine the conversion factor relating the two means, so as to provide a basis for the assessment of relatively actual exercise capacity of post-PCI patients and the formulation of accurate exercise prescriptions, thereby improving the safety of exercise prescription.

## Materials and Methods

### Subjects

A total of 41 patients were selected from those admitted to the Cardiac Rehabilitation Clinic, Chinese PLA General Hospital, from July 2015 to June 2016. Inclusion criteria include chronic coronary syndrome confirmed by coronary arteriography, status-post-stent implantation. Exclusion criteria were as follows: (1) acute myocardial infarction (within 2 days); (2) high-risk unstable angina pectoris; (3) uncontrolled symptomatic arrhythmia or hemodynamic instability; (4) decompensated symptomatic heart failure; and (5) mental or physical handicaps or inability to cooperate during exercise.

The study was approved by the ethics committee of the Chinese PLA General Hospital and registered in Chinese clinical trial registry (ChiCTR2000031543). All subjects gave written informed consent. The whole study conforms with the principles outlined in the Declaration of Helsinki.

### Clinical Data Collection and Preparation

We established a clinical database to record basic information such as age, sex, height, and weight of all subjects. We calculated the body mass index (BMI) and waist-hip ratio (WHR) and recorded in detail the time of PCI treatment, the number of stents implanted, coexisting diseases such as hypertension, diabetes, and hyperlipidemia, and medications such as β-blockers, angiotensin-converting enzyme inhibitors/angiotensin receptor blockers (ACEI/ARB), statins, diltiazem, and nitrates. Patients were asked not to eat within 3 h before starting the exercise testing; however, they could drink an appropriate amount of water. The subjects were asked to wear comfortable and suitable clothes, shoes, and socks. Other strenuous exercise was to be avoided within 24 h of exercise testing, and medications such as β-blockers, calcium antagonists, and nitrates should be held until immediately after the testing. All patients underwent CPET first, and then TET 1 week later, from which the relevant results were recorded.

### Cardiopulmonary Exercise Testing

A cycle ergometer (CS-200, Schiller, Obfelden, Switzerland) was adopted as the exercise device. Calibration was performed before each testing. The calibrated gases were as follows: 4% CO_2_, 16% O_2_, and N_2_ (the balance). A ramp protocol was applied to identify the symptom-limiting maximum exercise load. The specific protocol was as follows: 0 W: rest for 1 min; 0 W: warm-up for 2 min; the treadmill intensity started at 5 W. Thereafter, the intensity for men was ramped up at 25 W/min and for women it was 20 W/min. The speed was maintained at 60–70 rpm until the maximum exercise load was reached (i.e., no more exercise could be performed because of dyspnea or fatigue of the legs or the whole body, or rotational speed decreased to <50 rpm). The recovery phase intensity was 0 W until the heart rate, VO_2_, VCO_2_, and other indicators returned to baseline, whereupon testing was terminated. The measured METs was calculated using the following equation: METs = peak VO_2_/3.5.

### Treadmill Exercise Testing

Submaximal exercise testing was carried out according to the Bruce protocol using the exercise treadmill (T2100, GE Medical Systems, Milwaukee, WI, USA). The specific protocol began at a gradient of 10% and a speed of 1.7 mph. At the end of each stage, the gradient increased by 2%; the speed increased to 2.5, 3.4, 4.2, 5.0, and 5.5 mph for the subsequent stages. The patient exercised at each grade for 3 min until the maximum exercise load was reached, then entered the recovery state and gradually decreased to grade I, grade 0, and warm-up (1.6 mph, 0%), with each grade being 2 min apart. The testing was terminated when heart rate, VO_2_, VCO_2_, and other indicators return to baseline. The patient's subjective level of exertion was qualified using the Borg 6–20 scale which is a simple method of rating perceived exertion (RPE) (6 means about 20% while 20 means exhaustion). The MET value was estimated by a software (GE Cardiac Assessment System for Exercise Testing, Milwaukee, WI, USA) using the following equation: METs = [3.5 + 26.8 * 0.1 * (speed in mph) + (speed in mph) * 26.8 ^*^ (% gradient) * 1.8]/3.5.

### Statistical Methods

Statistical analysis was performed using SPSS version 23.0(IBM SPSS Statistics for Windows, New York, USA). Quantitative variables were first tested for normality. Those following normal distribution were expressed as mean ± standard deviation (*x* ± *s*), whereas those that were not were expressed as median and interquartile range. The qualitative data were expressed as ratio or percentage. The paired *t*-test was used to compare the indicators of the two tests in the same patient. *p* < 0.05 determined statistical significance. Finally, count data were graded/classified, assigned values, and then converted into binary variables for statistical analysis (while excluding outliers) and multiple linear regression analysis. The variables were screened using a stepwise screening method.

## Results

### General Clinical Data

**Figure 1:**
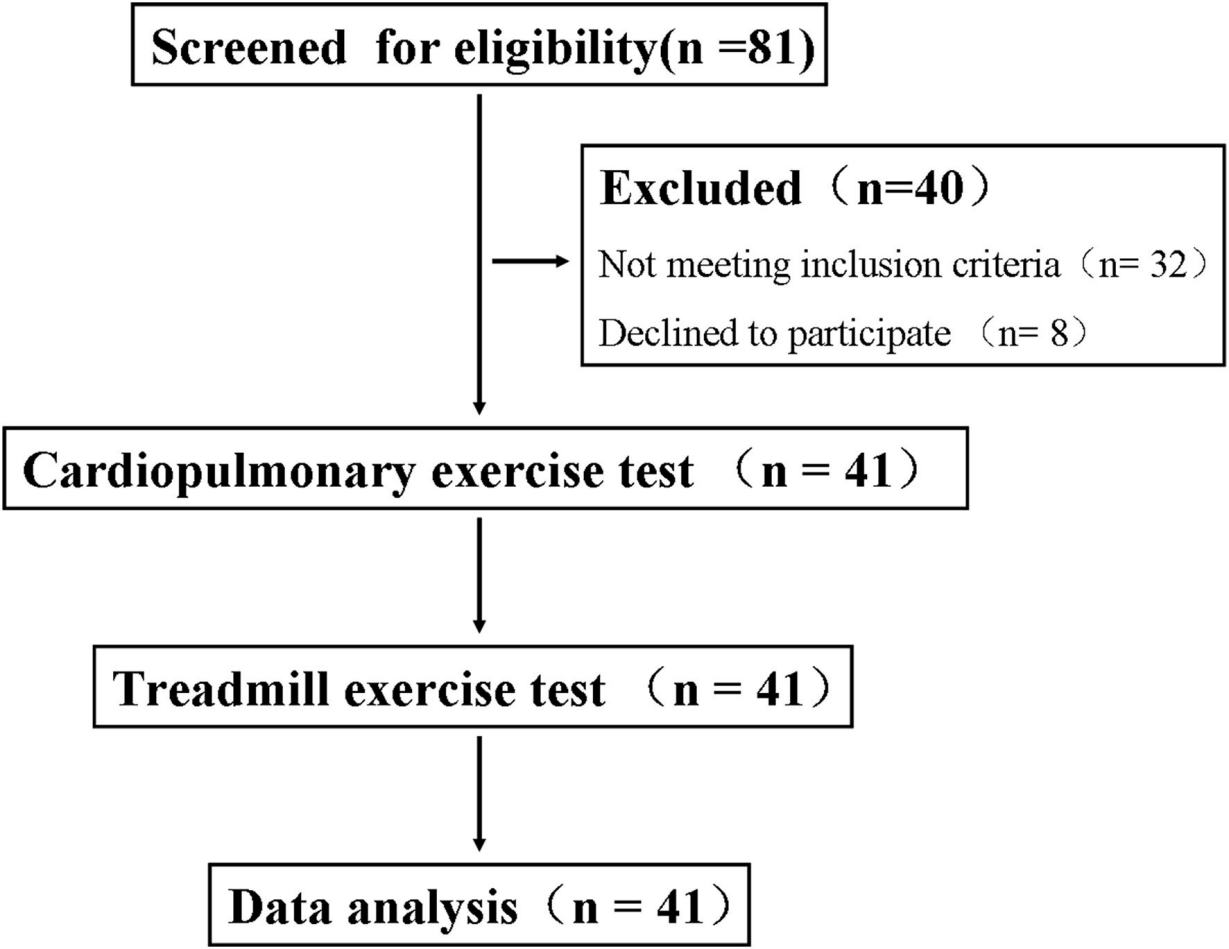
Flow diagram of the participants.

A total of 41 subjects were enrolled in the study (Figure 1), patient characteristics are displayed in Table 1. All patients had undergone PCI revascularization before, of which 25 (61.0%) were due to acute myocardial infarction and 16 (39.0%) were due to unstable angina pectoris. The median number of stents implanted was 2 (1–4), and the median time between CPET and the last PCI was 6 (2.25–12 weeks). None of the patients had any discomfort such as chest pain 2 weeks before the test. in addition, biochemical and cardiac ultrasound indicators indicated that the disease was in a stable state.

**Table 1:** Subject characteristics (*n* = 41).

**Variables**	***Mean* ±*SD* or median (P25, P75) or percentage**
Male	34 (82.9)
Female	7 (17.1)
Age (years)	56.2 ± 9.2
Height (cm)	169.4 ± 7.6
Weight (kg)	75.3 ± 11.1
BMI (kg/m^2^)	26.2 ± 2.9
Waist-hip ratio	0.78 ± 0.36
Smokers (%)	14 (34.1)
Previous myocardial infarction	25 (61.0)
**No. of stenotic coronary arteries**	
1	29 (70.7)
2	8 (19.5)
3	4 (9.8)
No. of stents	2 (1,4)
The time between CPET and the latest PCI (weeks)	6 (2.25, 12)
**Coexisting disease**	
Hypertension	25 (61.0)
Diabetes	11 (26.8)
Hyperlipidemia	26 (63.4)
**Medication**	
β-Blocker	27 (65.7)
ACEI/ARB	11 (26.8)
Stain	38 (92.7)
Diltiazem	4 (9.8)
Nitrates	20 (48.8)
**Biochemical indicators** (reference range)	
BNP (pg/ml)	270.1 ± 275.7 (0–150)
Cr (μmol/L)	80.7 ± 16.9 (30–110)
TnT (ng/L)	0.02 ± 0.03 (0–0.1)
CK-MB (ng/ml)	3.89 ± 4.19 (0–6.5)
TG (mmol/L)	3.78 ± 0.93 (3.1–5.7)
LDL (mmol/L)	2.21 ± 0.74 (0–3.4)
**Cardiac ultrasound indicators** (reference range)	
LVEF (%)	58.1 ± 7.6 (50–70)
LVIDd (mm)	46.3 ± 4.6 (37–53)
IVSd (mm)	10.7 ± 1.3 (8–11)
Regional wall motion	14 (34.1)

*BMI, body mass index; ACEI/ARB, angiotensin converting enzyme inhibitor/angiotensin receptor blocker; LVEF, left ventricular ejection fraction; LVIDd, left ventricular diastolic dimension; IVSd, interventricular septal thickness*.

### Comparison of the Termination of Treadmill Exercise Testing and Cardiopulmonary Exercise Testing

All 41 subjects completed CPET and TET. During the interval between the two tests, no patients had angina pectoris attacks or respiratory symptoms. In the TET, all patients stopped the testing after reaching the target heart rate, of which 87.8% (36/41) had negative results, 7.3% (3/41) had suspiciously positive results, and 4.9% (2/41) had positive results. In the CPET, 97.6% (40/41) of the 41 subjects terminated the testing because of fatigue, and very few terminated the testing because of chest distress (2.4%, 1/41). During the testing, only one patient had ST-segment changes (2.4%, 1/41). At the end of the testing, the mean Brog score of the subjects was 15.08, and the mean maximum amount of exercise reached 5.19 METs.

### Comparison of Exercise Capacity Measured in Treadmill Exercise Testing and Cardiopulmonary Exercise Testing

**Figure 2:**
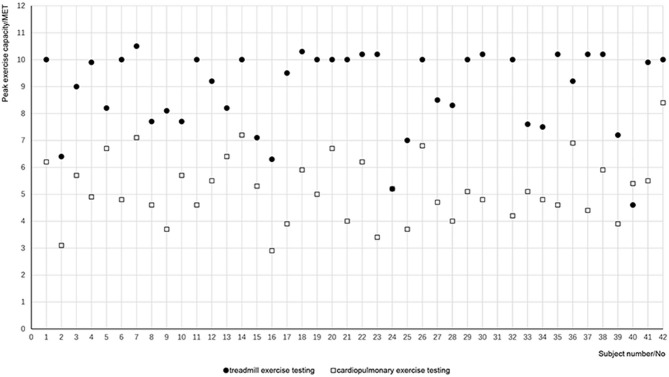
Exercise capacity measured by the two tests for each patient. Solid dots refer to the METs calculated by treadmill exercise test for each patient, and open squares refer to the METs measured by cardiopulmonary exercise test.

The subjects showed a mean maximum exercise capacity of 8.89 METs during the testing (Table 2). There was no significant difference in maximum heart rate during the two testing (*p* = 0.842); however, there was a significant difference in the maximum exercise capacity (*p* = 0.000). Figure 2 lists the maximum exercise capacity of all 41 patients. It can be seen that the maximum exercise capacity measured by TET was generally greater than that of CPET. The percentage of the METs measured by CPET to that estimated by TET averaged 59.85 ± 2.61%, with a confidence interval of 54.56–65.13%.

**Table 2:** Comparison of the two kinds of exercise testing.

	**CPET**	**TET**	***t*-Value**	***p*-Value**
METmax	5.19 ± 1.23	8.89 ± 1.53	14.51	0.000
HRmax	126.22 ± 21.16	127.11 ± 13.08	−0.20	0.842
HRrest	70.56 ± 9.83	74.75 ± 12.98	1.98	0.056
ΔSBP	53 ± 19	39 ± 19	−3.15	0.003
ΔDBP	9 ± 14	11 ± 32	3.54	0.001

*CPET, cardiopulmonary exercise testing; TET, treadmill exercise testing; MET, metabolic equivalent; HR, heart rate; SBP, systolic blood pressure; DBP, diastolic blood pressure*.

### Factors Affecting the Difference in METs

The exercise capacity measured in the CPET was less than that of the TET, and the percentage of the difference in terms of treadmill exercise capacity averaged 40.15 ± 2.61%. Multiple linear regression analysis of the factors affecting the difference suggested that the difference of METs was related to WHR but was not significantly associated with age, sex, BMI, β-blockers, ACEI/ARB, statins, diltiazem, or nitrates (Table 3). The specific relationship is shown in the following regression equation:

$$\begin{array}{c} \text{Y}1=-1.591\text{X}1+\;5.088 \end{array}$$

where *Y*1 represents the maximum MET difference in the exercise capacity between the two testing, and *X*1 represents the WHR. From the equation, it can be seen that there is a negative correlation between MET difference and WHR.

**Table 3:** Regression estimation 1.

**Model**	**B**	**Std. Error**	**β**	***t***	**Sig**.
Constant	5.088	0.640		7, 953	0.000
Waist-hip ratio	−1.591	0.739	0.356	−2.153	0.039

When the dependent variable is changed to the percentage of MET difference in terms of treadmill exercise capacity, and the independent variable remains unchanged, the results of multiple linear regression analysis were similar to those of the previous results. The percentage of MET difference in treadmill exercise capacity was negatively associated with WHR; however, it was not significantly associated with age, sex, BMI, β-blockers, ACEI/ARB, statins, diltiazem, or nitrates (Table 4). The specific relationship is shown in the following equation:

$$\begin{array}{c} \text{Y}2=-16.244\text{X}2+\;54.847 \end{array}$$

where *Y*2 is the percentage of MET difference in treadmill exercise capacity, and *X*2 is WHR.

**Table 4:** Regression estimation 2.

**Model**	**B**	**Std. Error**	**β**	***t***	**Sig**.
Constant	54.847	5.882		7, 953	0.000
Waist-hip-rate	−16.244	6.796	−0.389	−2.153	0.023

## Discussion

The present study is the first of its kind to measure the conversion factor between exercise capacity in post-PCI patients using CPET and TET, and we got two functions to estimate a more accurate MET by TET result.

We found a significant difference in maximum exercise capacity between CPET and TET in the same subject within 1 week. The estimated value in TET is likely to be greater than the actual value measured in CPET; in our study, the measured MET averaged (59.85 ± 2.61%) of the estimated value. Recent evidence demonstrated exercise capacity plays an important role for the prognosis of obviously healthy people or ill people. While 1 MET increase in exercise capacity is considered to decrease the risk of all-cause mortality by 13% (15). Reasonable CR exercise training not only improves the mobility and quality of life, but it also improves long-term prognosis and reduces the probability of recurrent cardiovascular events (16, 17). Cao et al. (18) demonstrated that only a certain intensity of exercise could improve the relevant parameters before and after CR. When an exercise plan is formulated, the exercise intensity is usually set to 50–80% of the patient's maximum exercise capacity (19). Low-intensity exercise cannot achieve the goal of improving cardiac function, whereas high-intensity exercise may exceed the adaptation range of the patient's cardiorespiratory fitness (20). Estimation by the results of simple TET may lead to overestimation of actual exercise capacity and therefore may incorrectly match rehabilitation training intensity with actual cardiac function, thereby increasing cardiovascular risk in cardiac rehabilitation training.

The results of this study are consistent with those of previous studies. The maximum exercise capacity measured by TET is higher than that of CPET using a cycle ergometer as a dynamometer (8). The stepwise power-increasing Bruce protocol is commonly adopted in TET, while the increasing ramp protocol is commonly adopted in CPET. According to Myers' study, whether using treadmill or cycle ergometer, there was a significant difference in exercise capacity in the same patient between a stepwise increasing exercise program and a ramp increasing exercise program (12, 21). The amount of exercise is thought to be proportional to exercise time, which is the basis of the estimation formula of METs. However, the increment of a stepwise increasing exercise program is incontinuous actually. Post-PCI patients often suffer from cardiac insufficiency; as a consequence, their actual ejection fraction cannot increase linearly with the amount of exercise (20, 22, 23). Nevertheless, the estimation of the exercise capacity in TET is based on a linear formula. Therefore, for post-PCI patients, exercise capacity estimated by TET may be higher than the actual value (24). Another possible explanation might involve the effecting muscles. Cycle ergometer exercise is more dependent on lower limb muscles, while the handrails equipped with TET may produce additional resistance and allocate more muscle to work (25).

We found that the average percentage of the difference in the METs estimated by TET was 40.15%, (95% confidence interval: 35.03–45.27%). Multiple linear regression analysis showed yielded a function: *Y*1 = −1.591*X*1 + 5.088 (*Y*1 represents the maximum MET difference in the exercise capacity between the two testing, *X*1 represents the WHR). Considering the MET by TET was an estimated value, we changed the dependent variable to the percentage of MET difference in terms of treadmill exercise capacity and got a new function: *Y*2 = −16.244*X*2 + 54.847. These two functions indicate that the difference and the difference in the METs estimated by TET both negatively correlated with the WHR. A study that attempted to compare measured and estimated METs in TET found that the difference had a weak positive correlation to age (26). Considering that the main purpose of this study was to improve the accuracy of diagnosis, the subjects of the study were all patients with heart-related symptoms while the subjects of our study were all patients after PCI, which may result in the difference between the results. Some drugs routinely used after PCI, such as β-blockers and calcium channel blockers, may affect exercise capacity (27, 28). All drugs were temporarily suspended 24 h before the exercise testing; nevertheless, we included them among the independent variables for multivariate linear analysis. We found use of these medications had no bearing on the difference between the two testing methods or on the percentage of the difference in the METs estimated by TET.

Given that gas measurements and analysis devices are not common in developing countries, it is more feasible to estimate exercise capacity using TET; however, we suggest that the results of TET be reduced by 35–45% to be used for the consequent exercise prescription. Previous studies have indicated that self-assessment questionnaires such as the Duke Activity Status Index and the Veterans Specific Activity Questionnaire can provide a rough assessment of exercise capacity through questionnaires regarding daily activity capacity of various magnitudes (4, 29). The combination of the two estimation methods may improve the safety of exercise training and the accuracy of exercise prescription.

There are limitations in the present study. Although the study demonstrated significant different effects between two exercise tests, the sample size was relatively small and involved only a single center. A multicenter clinical trial with larger sample size is on the way to further explore the differential evaluating effect on exercise capacity of CPET and TET in post-PCI patients.

## Conclusion

The basis of reasonable cardiac rehabilitation training for post-PCI patients is correct assessment of exercise capacity. Even though current international guidelines recommend CPET, when CPET or other metabolic testing were not available, exercise capacity can be estimated indirectly using simple TET, the results of which should be reduced by 35–45%. Two formulas could also be used to attain a more accurate METs. The results of this study might supply a more accurate means of evaluating the exercise capacity for the post-PCI patients when CPET is not available.

## Data Availability Statement

The original contributions presented in the study are included in the article/supplementary materials, further inquiries can be directed to the corresponding author/s.

## Ethics Statement

The studies involving human participants were reviewed and approved by the ethics committee of the Chinese PLA General Hospital. The patients/participants provided their written informed consent to participate in this study.

## Author Contributions

The study was initiated by JM and YX. YG and BF performed the statistical analysis and drafted the manuscript. RH, YZ, and YS were helpful for data collection. JM and YX contributed substantially to its revision and took responsibility for the manuscript as a whole. All authors contributed to the article and approved the submitted version.

## Conflict of Interest

The authors declare that the research was conducted in the absence of any commercial or financial relationships that could be construed as a potential conflict of interest.

## Publisher's Note

All claims expressed in this article are solely those of the authors and do not necessarily represent those of their affiliated organizations, or those of the publisher, the editors and the reviewers. Any product that may be evaluated in this article, or claim that may be made by its manufacturer, is not guaranteed or endorsed by the publisher.
